# Dataset of vehicle images for Indonesia toll road tariff classification

**DOI:** 10.1016/j.dib.2020.106061

**Published:** 2020-07-23

**Authors:** Ananto Tri Sasongko, Grafika Jati, Mohamad Ivan Fanany, Wisnu Jatmiko

**Affiliations:** a Faculty of Computer Science, Universitas Indonesia, Indonesia

**Keywords:** Dataset, Vehicle image, Deep learning, Classification, Transfer learning, Fine-tuning

## Abstract

Vehicle classifications with different methods have been applied for many purposes. The data provided in this article is useful for classifying vehicle purposes following the Indonesia toll road tariffs. Indonesia toll road tariff regulations divide vehicles into five groups as follows, group-1, group-2, group-3, group-4, and group-5, respectively. Group-1 is a class of non-truck vehicles, while group-2 to group-5 are classes of truck vehicles. The non-truck class consists of the sedan, pick-up, minibus, bus, MPV, and SUV. Truck classes are grouped based on the number of truck's axles. Group-2 is a class of trucks with two axles, a group-3 truck with three axles, a group-4 truck with four axles, and a group-5 truck with five axles or more. The dataset is categorized into five classes accordingly, which are group-1, group-2, group-3, group-4, and group-5 images. The data made available in this article observes images of vehicles obtained using a smartphone camera. The vehicle images dataset incorporated with deep learning, transfer learning, fine-tuning, and the Residual Neural Network (ResNet) model can yield exceptional results in the classification of vehicles by the number of axles.

Specifications Table

**Table:** 

Subject	Computer Vision and Pattern Recognition
Specific subject area	Vehicle Image and Classification
Type of data	2-Dimensional 24-bit RGB Color Image
How data were acquired	By taking pictures and videos using a smartphone camera
Data format	Raw digital image (.jpg)
Parameters for data collection	The vehicles’ left or right side bodies were photographed uprightly to observe the number of wheels wholly.
Description of data collection	The vehicle pictures were collected by taking pictures and videos on the road, parking lot, and bus station. Data in the video formats were then converted to still images.
Data source location	Jakarta Area, Indonesia
Data accessibility	Name of Repository: Mendeley Data Dataset Location: https://data.mendeley.com/datasets/9s33gv3gbt/draft?a=d664b4e6-b18b-4bc9-b5e9-e45363f2bb79
Related research article	A. T. Sasongko and M. Ivan Fanany, "Indonesia Toll Road Vehicle Classification Using Transfer Learning with Pre-trained Resnet Models," 2019 International Seminar on Research of Information Technology and Intelligent Systems (ISRITI), Yogyakarta, Indonesia, 2019, pp. 373–378. https://doi.org/10.1109/ISRITI48646.2019.9034590 [1]

## Value of the data

• This dataset can be used to train deep learning models that can classify, detect and segment vehicles based on the number of wheels or axles since the images show the wheels wholly.
• Researchers interested in classifying, identifying and segmenting vehicles may use this vehicle image data, integrate it with data sets of other sources, and evaluate it for more insight.
• The data is comprehensive, containing five groups of vehicles (non-truck, 2-axle truck, 3-axle truck, 4-axle truck, and 5-axle truck).
• This dataset can be used to develop a new deep learning architecture using transfer learning techniques or modifying the existing one to improve the performance of the vehicle classification.

## 1 Data description

**Fig. 1:**
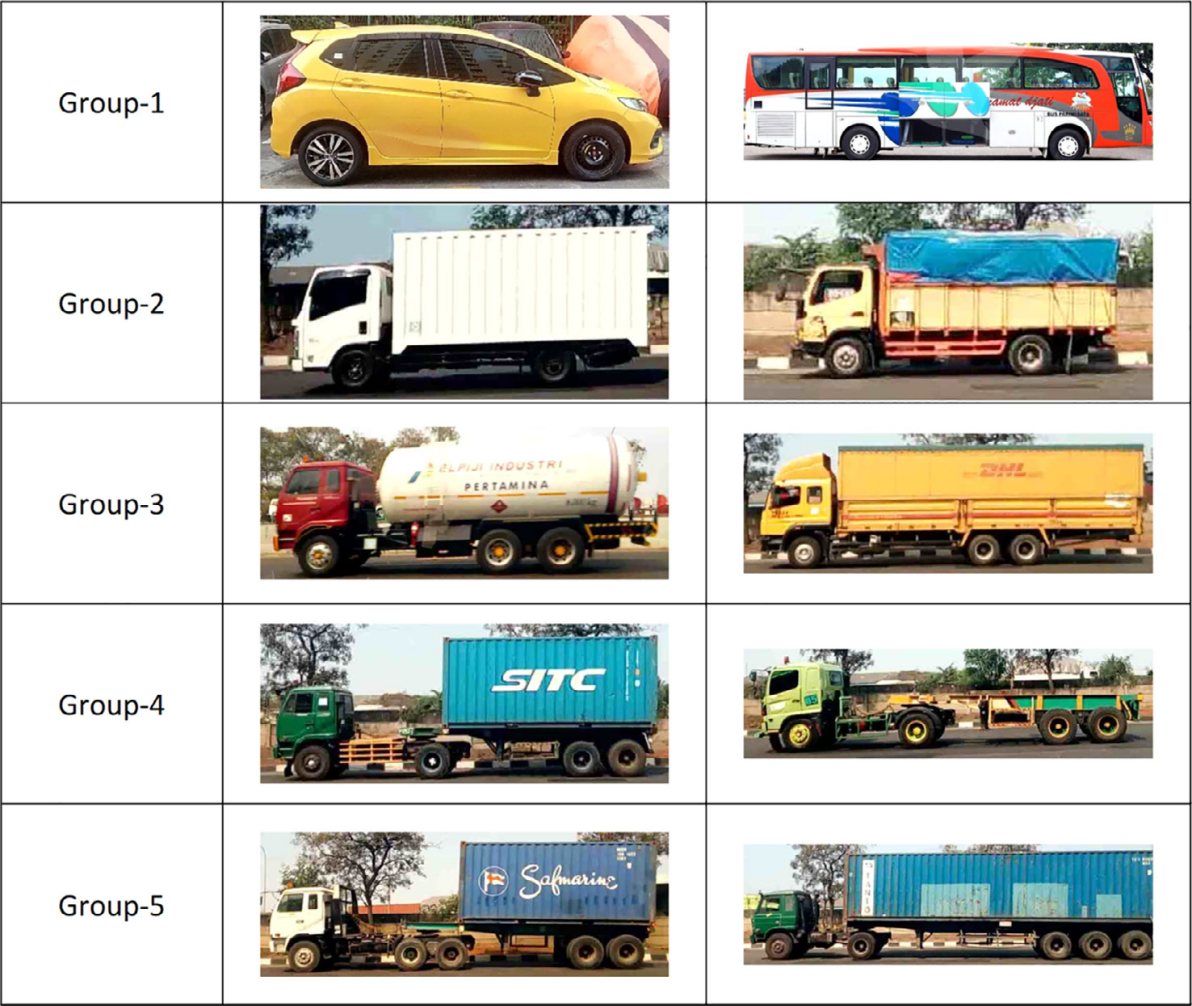
Samples of vehicle classification image dataset.

The vehicle images were gathered by taking pictures and video using a smartphone camera. The data in video formats were then converted to still images utilizing an *FFmpeg* command-line tool [2]. The data collected include the images of the car, sedan, van, pick-up, bus, minibus, truck, trailer truck, dump truck, garbage truck, and tanker truck. The dataset consists of 1200 images in JPG format with an image width of 512 pixels and various heights. The height varies depending on the size of the object being observed. The images are categorized into five classes, which are non-truck (group-1), 2-axle truck (group-2), 3-axle truck (group-3), 4-axle truck (group-4) and 5-axle or more truck (group-5). The non-truck class consists of the sedan, pick-up, minibus, bus, MPV, and SUV. Truck classes are grouped based on the number of truck's axles. Two-axle trucks are classified as group-2, three-axle trucks as group-3, four-axle trucks as group-4, and five or more axle trucks as a group-5. The number of images in each class is shown in Table 1. The data samples are presented in Fig. 1.

**Table 1:** The five vehicle classes and the number of images.

No	Category	Group	No. of Images
1	Non-Truck	1	400
2	2-Axle Truck	2	200
3	3-Axle Truck	3	200
4	4-Axle Truck	4	200
5	≥ 5-Axle Truck	5	200
Total			1200

## 2 Experimental design, materials and methods

**Fig. 2:**
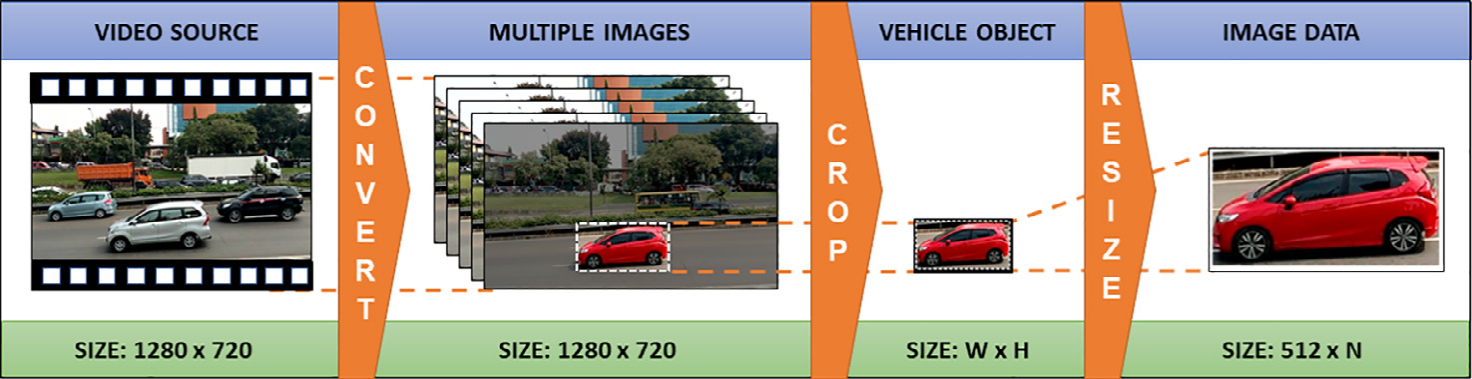
Conversion of the dataset from the video source to image data.

The vehicle dataset publicly available generally does not show the wheels wholly. Since the wheels are not clearly seen, it is difficult to count the number of axles. To know the axles, the vehicles’ left or right side bodies were necessarily photographed uprightly so the wheels can be observed thoroughly. The vehicle's axle is a component on which the left and right wheels are mounted. Knowing the wheels enables us to count the axles. The vehicle images dataset was collected by taking pictures and videos using a smartphone camera on the roads, parking lots, and bus stations in the Jakarta area. The smartphone camera specifications are 1280 × 720 pixels (720p) for videos in * .3gp format and 2560 × 1440 pixels for photos in * .jpg format. The data in video formats were then converted to still images using an *FFmpeg* command-line tool [2]. The dimension of converted images is 1280 × 720 pixels. Next, the original images were cropped around the vehicle object to minimize the background, then resized to 512 pixels wide and various heights to maintain the shape of the object by preserving the aspect ratio - this is useful for proper classification [3]. The conversion of the dataset from the video source to image data is shown in Fig. 2. Object cropping was undertaken manually using Microsoft Picture Manager. The dimension of the cropped object is not fixed, because it depends on the size of the vehicle object on the image derived by the video source. In general, the dimensions of the cropped image are W x H, where W stands for width and H for height. The unessential, unneeded images were deleted. After the refinement of the dataset, the number of vehicle images was reduced to 1200 images. The images are classified into five groups as follows: non-truck (group-1), 2-axle truck (group-2), 3-axle truck (group-3), 4-axle truck (group-4) and 5-axle or more truck (group-5).

Preparation of data is essential for classifying vehicles based on the concept of deep learning. When the dataset is limited, data augmentation strategy is required for multiplying training images in order to increase the accuracy [4]. The data augmentation methods are widely available in the current software framework for deep learning with a real-time image augmentation process [5], for this reason, the augmentation images are not provided in this vehicle dataset.

This dataset was used for deep learning-based vehicle classification by applying the transfer learning strategy and pre-trained ResNet models [1]. When the dataset is on a limited scale, a combination of pre-trained ResNet and transfer learning can speed up the result and gain higher performance compare to the conventional method [6]. Indeed, it improves predictions and exceeds baseline methods. Besides, the results can be significantly improved by applying fine-tuning [4].

## Declaration of Competing Interest

The authors declare that they have no known competing financial interests or personal relationships which could have influenced the work reported in this article.

## Appendix Supplementary materials

Image, application 1
